# Supplementary material for: Making MALDI-TOF MS for entomological parameters accessible: A practical guide for in-house library creation
Source: PLoS One. 2025 Aug 25;20(8):e0330605. doi: 10.1371/journal.pone.0330605 (PMC12377598; doi:10.1371/journal.pone.0330605)
Supplement: S1 File — This PDF contains the detailed step-by-step protocol for constructing MALDI-TOF MS reference database for insect identification. (PDF) [file pone.0330605.s001.pdf]

Apr 16, 2025

MALDI-TOF MS library development for insects

DOI
dx.doi.org/10.17504/protocols.io.n2bvjdy3xvk5/v1

Jonathan Karisa[1], Mercy Tuwei[1], Kelly Ominde[1], Brian Bartilol[1], Zedekiah Ondieki[1], Haron Musani[1], Martin Rono[1], Charles Mbogo[1], Philip Bejon[1], Joseph Mwangangi[1], Caroline Wanjiku[1], Marta Maia[1]

[1]Kenya Medical Research Institute, Wellcome Trust Research Program, Kilifi, Kenya

Philip Bejon: University of Oxford, Centre for Global Health and Tropical Medicine, Oxford, U.K
Marta Maia: 2Pwani University, Kilifi, Kenya; University of Oxford, Centre for Global Health and Tropical Medicine, Oxford, U.K

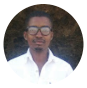

Jonathan Karisa
Kenya Medical Research Institute, Wellcome Trust Research Pr...

OPEN ACCESS

DOI: dx.doi.org/10.17504/protocols.io.n2bvjdy3xvk5/v1

External link: https://youtu.be/btS0hsx9yNw

Protocol Citation: Jonathan Karisa, Mercy Tuwei, Kelly Ominde, Brian Bartilol, Zedekiah Ondieki, Haron Musani, Martin Rono, Charles Mbogo, Philip Bejon, Joseph Mwangangi, Caroline Wanjiku, Marta Maia 2025. MALDI-TOF MS library development for insects. **protocols.io** https://dx.doi.org/10.17504/protocols.io.n2bvjdy3xvk5/v1

Manuscript citation:
1. Karisa J, Ominde K, Tuwei M, Bartilol B, Ondieki Z, Musani H, et al. Utility of MALDI-TOF MS for determination of species identity and blood meal sources of primary malaria vectors on the Kenyan coast. Wellcome Open Res. 2024;8: 151
2. Jonathan K. Developing mass spectrometry tools as a surveillance tool for vector control interventions. PhD Thesis, The Open University. 2024. Available: https://oro.open.ac.uk/99853/

License: This is an open access protocol distributed under the terms of the **Creative Commons Attribution License**, which permits unrestricted use, distribution, and reproduction in any medium, provided the original author and source are credited

Protocol status: Working
We use this protocol and it's working

Created: March 29, 2025

Last Modified: April 16, 2025

Protocol Integer ID: 125715

Funders Acknowledgements:
Bill and Melinda Gates Foundation
Grant ID: Investment 057212

Disclaimer

N/A

Abstract

Matrix-assisted laser desorption-ionisation time of flight mass spectrometry (MALDI-TOF MS) is a powerful analytical method that has been used extensively to identify sample ions of complex mixtures, and biological samples such as proteins, tissues and microorganisms. MALDI-TOF MS has revolutionised clinical microbiology with accurate, rapid, and inexpensive species-level identification of microbes. MALDI-TOF MS technology generates spectral signatures and matches them to a library of similar organisms using bioinformatics pattern matching. The use of MALDI-TOF MS for entomological samples has been explored by multiple groups with proven efficacy at differentiating between closely related species, as well as detecting pathogens in different vectors. The low cost per sample processing, rapid turnaround and robustness are attractive for surveillance of vector control programs. Libraries are built in-house for institutional usage, although a multi-user platform with sharing of spectra and data would be attractive, it is not yet available. Here, we outline a stepwise approach for creating an in-house MALDI-TOF MS library and subsequent query,
using malaria vector species identification as a case study for entomological samples.

Attachments

PDF — MALDI TOF MS library... 987KB

PNG — Figure 1. Sample pro... 81KB

PDF — Figure 2 Summary of ... 371KB

Image Attribution

N/A

Materials

Equipment, Materials and Reagent

Polymerase chain reaction

- Biosafety cabinet
- Clean room
- Centrifuge
- Thermocycler
- Gel imaging doc
- Electrophoresis tank
- Gel molders
- Flasks
- Ice and ice bucket
- Pipettes
- Pipette tips
- Sterile microcentrifuge tube
- 96-well PCR plates
- Plate sealers
- GoTaq® DNA Polymerase
- Primers
- Nuclease freeee water
- Staining solution (e.g Red safe)
- DNA ladder
- Loading dye
- Agarose
- TBE Buffer

Sanger sequencing

- MicroAmp Optical 96-well Reaction Plate
- BigDye Terminator v3.1 Cycle Sequencing Kit
- Ethanol
- EDTA
- NaOH
- Hi-Di Formamide

ELISA

- Phenol Red
- Igepal CA-630
- PBS
- Tween20
- NaOH
- HCl
- Glycerol
- Soft ELISA plates OR Hard ELISA plates
- Casein

- ABTS 2 component substrate

MALDI-TOF MS

- Deionized water
- Absolute ethanol (EtOH)
- Bacterial Test Standard (BTS)
- Acetonitrile (ACN)
- Formic Acid
- Trifluoracetic acid (TFA)
- Matrix HCCA, portioned
- Standard solvent (acetonitrile 50%, water 47.5% and trifluoroacetic acid 2.5%)
- 50 - 1000µl pipette tips and a suitable pipette
- 2- 200µl pipette tips and a suitable pipette
- 0.5 – 20µl pipette tips and a suitable pipette
- Microtube (Epperndorf safe- lock), PCR clean 1.5ml
- MALDI Target plate
- Benchtop centrifuge
- Vortex
- Ultrasonic bath

Mosquito sample processing in the laboratory

1 **Morphological identification**

1.1 Separate Culicine and Anopheles mosquitoes based on identification keys [1–3].

1.2 Identify female *Anopheles* mosquitoes at the species level using dichotomous morphological keys [2–4]

1.3 Sort each sample based on their physiological status (blood fed, unfed, gravid, and half gravid)

1.4 Assign a unique identifier to each sample and store under appropriate conditions

2 **Sample preparation**

2.1 Place an individual sample on clean white roll paper

2.2 Using a clean and sterile scalpel, dissect the mosquito into the following body compartments: legs and wings (L/W), Cephalothorax (Head and Thorax (HT)), and abdomen (ABD) (for blood-fed mosquitoes) **(Figure 1)**. Discard abdomens of unfed mosquitoes

2.3 Subdivide HT into two longitudinal halves.

2.4 Store the different compartments in separate microcentrifuge tubes labelled with the same identification code

2.5 One half of the HT is screened for *Plasmodium* infection status and species identification using PCR. Legs and wings are stored as backup samples if HT is used for *Plasmodium* detection using ELISA

3 **DNA extraction**

3.1 Extract DNA from the HT or L/W using the Chelex protocol as previously described [6,7]

4 **Species identification (cocktail PCR)**

4.1 Amplify the genomic DNA using *An. gambiae* s.l [8] and *An. funestus* s.l [9] species-specific diagnostic primers described by Scott et al (1993) and Koekemoer et al (2002) respectively.

4.2 Subject all the unamplified samples and those to be used for MALDI-TOF MS database creation to sequencing targeting ITS2 and COI regions as previously described by Karisa et al (2024) and Lobo et al (2015) [10,11]

5 ***Plasmodium* detection**

5.1 Use the CSP ELISA procedure outlined in the MR4 guidelines (2014)

5.2 Alternatively, use *Plasmodium* PCR assay such as those described by Echeverry et al (2017) or Snounou (1993 & 1995) [12–14]

MALDI-TOF MS analysisUntitled section

6 **Protein extraction and plate loading**

6.1 Add 15 µl of 70% (v/v) formic acid and 15 µl of 50% (v/v) acetonitrile (ACN) to the vial containing half of the head and thorax

6.2 Add a pinch of 106 µm acid wash glass beads until the glass beads are half full of the solvent in the vial

6.3 Homogenise sample in a Tissue Lyser II at 30Hz for 1 min for three cycles [10]

6.4 Centrifuge the samples at 13,000 rpm for 1 min to separate the debris from the proteins

6.5 Load one microliter (1 µl) of the sample on the MALDI-target plate in quadruplicate

6.6 Include one spot of Bacterial Test Standard (BTS) preparation as the positive control and four spots matrix only as the negative control

6.7 Allow the plate to air-dry at room temperature for 10-15 min

6.8 Overlay each sample spot on the plate with one µl of saturated matrix solution (composed of 0.25g α-cyano-4-hydroxycynnamic acid, acetonitrile (50% (v/v), trifluoroacetic acid (2.5% (v/v)) and 47.5% LC-MS grade water)

6.9 Allow the plate to dry again at room temperature for 10-15 min

7 Spectra acquisition

7.1 Load the plate into the Microflex instrument

7.2 Acquire spectra using the FlexControl software (Bruker Daltonics) with default settings or adjust the laser power (nitrogen laser) accordingly

7.3 Obtain spectra of mass ranges 2-20 kDa in a positive linear mode at a frequency of 60 Hz, an acceleration voltage of 20 kV, 200 ns extraction delay time, and a maximum laser power energy of 50%. Optimise the laser power until high quality spectra is achieved

7.4 Each spectrum is generated from 40 laser shots in six regions of the sample spot

8 **Spectra analysis**

8.1 Using FlexAnalysis software (Bruker Daltonics) [15], visually assess the spectra quality by checking general peak intensity (high intensity), the smoothness of the peaks, the flatness of the baseline and its reproducibility compared to other spectra of the same categories

8.2 Remove all the poor-quality spectra and flatlines from further processing

8.3 Load all the good quality spectra into MALDI Biotyper Explorer software (Bruker Daltonics) for processing, including smoothing, baseline subtraction, normalising, and peak selection

8.4 Perform cluster analysis using main spectrum profiles (MSP) dendrograms and composite correlation index (CCI) using MALDI-Biotyper v3.0 software to check the similarities between and among the spectra of the same and different categories

9 **Database creation using MALDI-Biotyper** [10,16]

9.1 Select the best quality spectra (based on an equitable algorithm on the MSP spectra' intensity, frequency, and peak position)

9.2 Select a minimum of ten spectra (from head and thorax) per mosquito sibling species with high spectral reproducibility to create the spectral database and increase discriminative power

9.3 Import the spectra in the MALDI-Biotyper software to create a reference database

10 **Database query, blinds tests and validation using MALDI-Biotyper 3.0.**

10.1 Select and load the MSP database of interest from the Taxonomy Tree View area

10.2 The MSPs are displayed on the MSP table, which can be used for classification or clustering

10.3 Load the rest of the spectra into the file view area of the MALDI-Biotyper 3.0 software

10.4 Select all the spectra and initiate the identification process by clicking the "*start identification*" button

10.5 The results are displayed on the "*Spectrum Scores table*", which summarises the results of a classification of an unknown spectrum with 10 top hits displayed

10.6 Use the spot with the highest log score value (LSV). The *MALDI* Biotyper produces *log score values* (LSV) ranging from 0.000 to 3.000 **(Table 1)**

10.7 For ambiguous results use the weighted log score value [15], and modify accordingly

10.8 Calculate a weighted LSV by multiplying the actual LSV by the inverse of their position and then by summing up the weighted LSV **(Figure 2)**

10.9 Compute the weighted LSV for each sample spotted in quadruplicate and calculate the mean of the weighted LSV for the four spots

10.10 The sibling species with the highest mean weighted LSV is considered the probable sibling species identity [10]

Protocol references

1. Edwards FW. Mosquitoes of the Ethiopian Region. HI.-Culicine Adults and Pupae. 1941 [cited 10 Feb 2025]. Available: https://www.cabidigitallibrary.org/doi/full/10.5555/19412901141
2. Gillies MT, Coetzee M. A supplement to the Anophelinae of Africa South of the Sahara. Publ Afr Inst Med Res. 1987;55: 1–143.
3. Gillies MT, De Meillon B. The Anophelinae of Africa south of the Sahara (Ethiopian zoogeographical region). 1968 [cited 10 Feb 2025]. Available: https://www.cabidigitallibrary.org/doi/full/10.5555/19692900946
4. Coetzee M. Key to the females of Afrotropical Anopheles mosquitoes (Diptera: Culicidae). Malar J. 2020;19: 70. doi:10.1186/s12936-020-3144-9
5. Jonathan K. Developing mass spectrometry tools as a surveillance tool for vector control interventions. PhD Thesis, The Open University. 2024. Available: https://oro.open.ac.uk/99853/
6. Karisa J, Ominde K, Muriu S, Munyao V, Mwikali K, Babu L, et al. Malaria vector bionomics in Taita-Taveta County, coastal Kenya. Parasit Vectors. 2022;15: 430. doi:10.1186/s13071-022-05527-w
7. Musapa M, Kumwenda T, Mkulama M, Chishimba S, Norris DE, Thuma PE, et al. A simple Chelex protocol for DNA extraction from Anopheles spp. J Vis Exp JoVE. 2013; 3281. doi:10.3791/3281
8. Scott JA, Brogdon WG, Collins FH. Identification of single specimens of the Anopheles gambiae complex by the polymerase chain reaction. Am J Trop Med Hyg. 1993;49: 520–529.
9. Koekemoer LL, Kamau L, Hunt RH, Coetzee M. A cocktail polymerase chain reaction assay to identify members of the Anopheles funestus (Diptera: Culicidae) group. Am J Trop Med Hyg. 2002;66: 804–811.
10. Karisa J, Ominde K, Tuwei M, Bartilol B, Ondieki Z, Musani H, et al. Utility of MALDI-TOF MS for determination of species identity and blood meal sources of primary malaria vectors on the Kenyan coast. Wellcome Open Res. 2024;8: 151.
11. Lobo NF, Laurent BS, Sikaala CH, Hamainza B, Chanda J, Chinula D, et al. Unexpected diversity of Anopheles species in Eastern Zambia: implications for evaluating vector behavior and interventions using molecular tools. Sci Rep. 2015;5: 17952.
12. Echeverry DF, Deason NA, Makuru V, Davidson J, Xiao H, Niedbalski J, et al. Fast and robust single PCR for Plasmodium sporozoite detection in mosquitoes using the cytochrome oxidase I gene. Malar J. 2017;16: 230. doi:10.1186/s12936-017-1881-1
13. Snounou G. Detection and Identification of the Four Malaria Parasite Species Infecting Humans by PCR Amplification. Species Diagnostics Protocols. New Jersey: Humana Press; 1995. pp. 263–292. doi:10.1385/0-89603-323-6:263
14. Snounou G, Viriyakosol S, Jarra W, Thaithong S, Brown KN. Identification of the four human malaria parasite species in field samples by the polymerase chain reaction and detection of a high prevalence of mixed infections. Mol Biochem Parasitol. 1993;58: 283–292.
15. Bruker Daltonik GmbH. flexAnalysis 3.4 User Manual. 2011.
16. Bruker Daltonik GmbH. MBT Explorer Module User Manual.
17. Harju I, Lange C, Kostrzewa M, Maier T, Rantakokko-Jalava K, Haanperä M. Improved Differentiation of Streptococcus pneumoniae and Other S. mitis Group Streptococci by MALDI Biotyper Using an Improved MALDI Biotyper Database Content and a Novel Result Interpretation Algorithm. Richter SS, editor. J Clin Microbiol. 2017;55: 914–922. doi:10.1128/JCM.01990-16

Acknowledgements

We are grateful to the Scientific and technical teams at the Centre for Geographic Medicine Research Coast, Kilifi for help in design and implementation of this work. Many thanks to the technical and field staff (Festus Yaa, Gabriel Nzai, Robert Mwakesi and Martha Muturi) who devoted their time and assisted in the field collection of samples.
