# Supplementary material for: Making MALDI-TOF MS for entomological parameters accessible: A practical guide for in-house library creation
Source: PLoS One. 2025 Aug 25;20(8):e0330605. doi: 10.1371/journal.pone.0330605 (PMC12377598; doi:10.1371/journal.pone.0330605)
Supplement: Attachment — Submitted filename: Response to Reviewers.docx [file pone.0330605.s003.docx]

**Comments and response**

1. Please ensure that your manuscript meets PLOS ONE's style requirements, including those for file naming.

The manuscript and associated figures and supplementary documents have been edited and aligned as per the PLOS ONE requirements

2. We note you have not yet provided a [protocols.io](http://protocols.io) PDF version of your protocol and/or a [protocols.io](http://protocols.io) DOI. When you submit your revision, please provide a PDF version of your protocol as generated by [protocols.io](http://protocols.io) (the file will have the [protocols.io](http://protocols.io) logo in the upper right corner of the first page) as a Supporting Information file. The filename should be S1_file.pdf, and you should enter “S1 File” into the Description field. Any additional protocols should be numbered S2, S3, and so on. Please also follow the instructions for Supporting Information captions [<https://journals.plos.org/plosone/s/supporting-information#loc-captions>]. The title in the caption should read: “Step-by-step protocol, also available on protocols.io.” Please assign your protocol a [protocols.io](http://protocols.io) DOI, if you have not already done so, and include the following line in the Materials and Methods section of your manuscript: “The protocol described in this peer-reviewed article is published on [protocols.io](http://protocols.io) ([https://dx.doi.org/10.17504/protocols.io.[...]](https://dx.doi.org/10.17504/protocols.io.%5B...%5D)) and is included for printing purposes as S1 File.” You should also supply the DOI in the Protocols.io DOI field of the submission form when you submit your revision.
   If you have not yet uploaded your protocol to [protocols.io](http://protocols.io), you are invited to use the platform’s protocol entry service [<https://www.protocols.io/we-enter-protocols>] for doing so, at no charge. Through this service, the team at [protocols.io](http://protocols.io) will enter your protocol for you and format it in a way that takes advantage of the platform’s features. When submitting your protocol to the protocol entry service please include the customer code PLOS2022 in the Note field and indicate that your protocol is associated with a PLOS ONE Lab Protocol Submission. You should also include the title and manuscript number of your PLOS ONE submission.

The step-by-step protocol of the process here briefly described, is also available on protocols.io ([dx.doi.org/10.17504/protocols.io.n2bvjdy3xvk5/v1](https://dx.doi.org/10.17504/protocols.io.n2bvjdy3xvk5/v1)) and is included as a supplementary file with this article

3. Thank you for stating the following financial disclosure: “MM - Investment 057212 - Bill and Melinda Gates Foundation – USA. MR - FLR_R1_190497 - The Royal Society FLAIR fellowship grant- UK”Please state what role the funders took in the study. If the funders had no role, please state: "The funders had no role in study design, data collection and analysis, decision to publish, or preparation of the manuscript." If this statement is not correct you must amend it as needed. Please include this amended Role of Funder statement in your cover letter; we will change the online submission form on your behalf.

The funders had no role in study design, data collection and analysis, decision to publish, or preparation of the manuscript

4. We noted in your submission details that a portion of your manuscript may have been presented or published elsewhere. “Utility of MALDI-TOF MS for determination of species identity and blood meal sources of primary malaria vectors on the Kenyan coast” in Wellcome Open Research (10.12688/wellcomeopenres.18982.2). “Please clarify whether this [conference proceeding or publication] was peer-reviewed and formally published. If this work was previously peer-reviewed and published, in the cover letter please provide the reason that this work does not constitute dual publication and should be included in the current manuscript.

Utility of MALDI-TOF MS for determination of species identity and blood meal sources of primary malaria vectors on the Kenyan coast” in Wellcome Open Research (10.12688/wellcomeopenres.18982.2) is a peer reviewed publication. However, the current submission is a methodology manuscript detailing how to build a MALDI-TOF MS library for entomological samples and explaining the various technical steps analytical steps supported by a protocol. Although we present a case study on mosquitoes, this manuscript is relevant to this working on different insect classes. We detail this approach using data from a previously published article on Afro tropical malaria vectors. The present submission differs in scope and objectives from what has been previously published earlier which demonstrated the method’s ability to identify vectors compared to gold standard. The earlier paper has been referenced. The data from the previous published work is shown in this manuscript only as a case study to elucidate how to build the libraries and employ this technology.

5. When completing the data availability statement of the submission form, you indicated that you will make your data available on acceptance. We strongly recommend all authors decide on a data sharing plan before acceptance, as the process can be lengthy and hold up publication timelines. Please note that, though access restrictions are acceptable now, your entire data will need to be made freely accessible if your manuscript is accepted for publication. This policy applies to all data except where public deposition would breach compliance with the protocol approved by your research ethics board. If you are unable to adhere to our open data policy, please kindly revise your statement to explain your reasoning and we will seek the editor's input on an exemption. Please be assured that, once you have provided your new statement, the assessment of your exemption will not hold up the peer review process.

All the data related to this publication has been deposited in the Harvard Dataverse. This includes the dataset, and MALDI-TOF MS Main Spectral Profiles (Libraries) for species identification (https://doi.org/10.7910/DVN/VYQFNO37)

Please review your reference list to ensure that it is complete and correct. If you have cited papers that have been retracted, please include the rationale for doing so in the manuscript text, or remove these references and replace them with relevant current references. Any changes to the reference list should be mentioned in the rebuttal letter that accompanies your revised manuscript. If you need to cite a retracted article, indicate the article’s retracted status in the References list and also include a citation and full reference for the retraction notice.

All the references are per as the PLOS one guidelines

**Additional Editor Comments:**

The procedures and results reported in this manuscript have largely been been published previously (<https://doi.org/10.12688/wellcomeopenres.18982.2>) and the added value of this article appears somewhat limited.

The current submission is a methodology manuscript detailing how to build a MALDI-TOF MS library for entomological samples and explaining the various technical steps analytical steps supported by a protocol. Although we present a case study on mosquitoes, this manuscript is relevant to this working on different insect classes. We detail this approach using data from a previously published article on Afro tropical malaria vectors. The present submission differs in scope and objectives from what has been previously published earlier which demonstrated the method’s ability to identify vectors compared to gold standard. The earlier paper has been referenced. The data from the previous published work is shown in this manuscript only as a case study to elucidate how to build the libraries and employ this technology.

In the abstract section, the authors wrote "Libraries are built in-house for institutional usage, although a multi-user platform with sharing of spectra and data would be attractive, it is not yet available." This sentence is erroneous and should revised, providing references to published studies that strived to develop a free identification service available online (<https://doi.org/10.1371/journal.pone.0305167>), a free open-source data analysis pipeline and share mass spectral data (<https://doi.org/10.1186/s13071-024-06655-1>).

The sentence has been revised to read “*Libraries are built in-house for institutional usage, although a multi-user platform with sharing of spectra and data would be attractive. Only a few studies have strived to make their libraries publicly available*”

Moreover, the MALDI-TOF MS Main Spectral Profiles (Libraries) for species identification related to this publication has been deposited in public repository - Harvard Dataverse (<https://doi.org/10.7910/DVN/VYQFNO37>)

I would also strongly recommend to the authors to deposit the mass spectra on a public repository to facilitate the widespread uptake of MALDI-TOF MS for mosquito species identification.

The MALDI-TOF MS Main Spectral Profiles (Libraries) for species identification related to this publication has been deposited in public repository i.e. the Harvard Dataverse (<https://doi.org/10.7910/DVN/VYQFNO37>) for any one with MALDI-TOF MS equipment to adapt and update for their use
