# Supplementary material for: Making MALDI-TOF MS for entomological parameters accessible: A practical guide for in-house library creation
Source: PLoS One. 2025 Aug 25;20(8):e0330605. doi: 10.1371/journal.pone.0330605 (PMC12377598; doi:10.1371/journal.pone.0330605)
Supplement: Attachment — Submitted filename: Response_to_Reviewers_auresp_2.docx [file pone.0330605.s004.docx]

**Comments and response**

1. If the reviewer comments include a recommendation to cite specific previously published works, please review and evaluate these publications to determine whether they are relevant and should be cited.

Response – In the previous review, the reviewers recommended two articles:

- (<https://doi.org/10.1371/journal.pone.0305167>), a free open-source data analysis pipeline and share mass spectral data
- (<https://doi.org/10.1186/s13071-024-06655-1>).

Upon reviewing these publications, we noted that the methods used differ from ours. Specifically, both studies employed R and Python scripts for post-acquisition spectral processing. In contrast, our study utilized the MALDI-Biotyper® software for spectral processing and analysis. None of these reference have been included in our manuscript. Regarding the recommendation to deposit mass spectra in a public repository, we confirm that all relevant data associated with this study have been deposited in the Harvard Dataverse. This includes both the raw dataset and the MALDI-TOF MS Main Spectral Profiles (MSPs) used for species identification. The data are accessible via the following DOI: <https://doi.org/10.7910/DVN/VYQFNO37>

2. Please review your reference list to ensure that it is complete and correct. If you have cited papers that have been retracted, please include the rationale for doing so in the manuscript text, or remove these references and replace them with relevant current references. Any changes to the reference list should be mentioned in the rebuttal letter that accompanies your revised manuscript. If you need to cite a retracted article, indicate the article’s retracted status in the References list and also include a citation and full reference for the retraction notice.

Response – Following your suggestion on the references cited in our manuscript, we conducted a thorough review of all the references included in the manuscript. We confirm that **none of the cited references appear to have been retracted,** and all remain accessible through their respective journals or digital repositories. To the best of our knowledge, all references are from **peer-reviewed and** meet PLOSOne journal requirements. If the editors or reviewers have any specific concerns regarding individual references, we would be happy to address them promptly.

3. The present submission demonstrated the method’s ability to identify vectors compared to gold standard. As the earlier paper has been referenced and used to show how to build the libraries and employ this technology. Authors should shorten the details shown in the current manuscript referring to other published work

Response – The manuscript, especially the methods section, has been revised and the relevant published work and step-by-step protocol deposited in protocol.io has been referenced.
